# Quality of interhospital transport of the critically ill: impact of a Mobile Intensive Care Unit with a specialized retrieval team

**DOI:** 10.1186/cc10064

**Published:** 2011-02-28

**Authors:** Janke S Wiegersma, Joep M Droogh, Jan G Zijlstra, Janneke Fokkema, Jack JM Ligtenberg

**Affiliations:** 1 Department of Critical Care (ICV), University Medical Center Groningen (UMCG), Hanzeplein 1, P.O. Box 30.001, 9700 RB Groningen, The Netherlands; 2 Intensive Care Unit, Scheper Hospital Emmen, Boermarkeweg 60, 7824 AA Emmen, The Netherlands

## Abstract

**Introduction:**

In order to minimize the additional risk of interhospital transport of critically ill patients, we started a mobile intensive care unit (MICU) with a specialized retrieval team, reaching out from our university hospital-based intensive care unit to our adherence region in March 2009. To evaluate the effects of this implementation, we performed a prospective audit comparing adverse events and patient stability during MICU transfers with our previous data on transfers performed by standard ambulance.

**Methods:**

All transfers performed by MICU from March 2009 until December 2009 were included. Data on 14 vital variables were collected at the moment of departure, arrival and 24 hours after admission. Variables before and after transfer were compared using the paired-sample T-test. Major deterioration was expressed as a variable beyond a predefined critical threshold and was analyzed using the McNemar test and the Wilcoxon Signed Ranks test. Results were compared to the data of our previous prospective study on interhospital transfer performed by ambulance.

**Results:**

A total of 74 interhospital transfers of ICU patients over a 10-month period were evaluated. An increase of total number of variables beyond critical threshold at arrival, indicating a worsening of condition, was found in 38 percent of patients. Thirty-two percent exhibited a decrease of one or more variables beyond critical threshold and 30% showed no difference. There was no correlation between patient status at arrival and the duration of transfer or severity of disease. ICU mortality was 28%. Systolic blood pressure, glucose and haemoglobin were significantly different at arrival compared to departure, although significant values for major deterioration were never reached. Compared to standard ambulance transfers of ICU patients, there were less adverse events: 12.5% vs. 34%, which in the current study were merely caused by technical (and not medical) problems. Although mean Acute Physiology and Chronic Health Evaluation II (APACHE II) score was significantly higher, patients transferred by MICU showed less deterioration in pulmonary parameters during transfer than patients transferred by standard ambulance.

**Conclusions:**

Transfer by MICU imposes less risk to critically ill patients compared to transfer performed by standard ambulance and has, therefore, resulted in an improved quality of interhospital transport of ICU patients in the north-eastern part of the Netherlands.

## Introduction

Transfer of critically ill patients in the Netherlands has recently been regulated by a national guideline and by law [1], prescribing a coordinating role for tertiary ICUs in different regions in the Netherlands. The emphasis of this more stringent regulation is coordination, consultation and if necessary, transferring the patient to a high intensity medically staffed ICU in order to facilitate a higher intensity of care or to give appropriate therapy.

Interhospital transport by standard ambulance is associated with limited monitoring capabilities and less staffed guidance during transfer than in the ICU environment; thus imposing additional risk to the ICU patient [2-5]. In the Dutch setting, transfer of the critically ill was performed by standard ambulance with an ambulance nurse and a driver, and was occasionally accompanied by an ICU nurse or a physician [2,6].

The considerations of Dutch intensivists whether to transport a critically ill patient were assessed by van Lieshout *et al. *[7]: the most important determinants were the quality of escorting personnel as well as the transport facilities. Neither characteristics of the patient's condition nor the level of supportive care seemed to be of significance in this process. The results of this study reflect the importance of a well established transporting device. The implementation of a MICU or a specialist retrieval team has been shown to be effective in reducing risks in other countries [8,9].

From March 2009 on, in order to conform to the national guidelines, a specially designed large-volume MICU and a specialized retrieval team, serving the region near our university medical center, have been used to transfer critically ill patients.

To find out to which extent incidents and adverse events happen during and shortly after these transfers, we conducted a prospective audit, including transfers performed by MICU from March until December 2009. In this observational study we tried to find an answer to the following questions: What is the relative frequency of events during transfer? What proportion of events is due to technical failure and/or to staff management? What is the influence of transfer and events on the condition of the critically ill patient; for example, did vital variables, documented before transfer, pass any critical threshold during transfer? What is the 24-hour ICU mortality rate (after transfer) and were there any significant factors that could predict such an outcome?

In order to evaluate the possible benefits of the MICU service, we compared results with data from hundred transfers done by standard ambulance transport in 2005 [2].

## Materials and methods

A stratified protocol clarification was sent to all referring ICUs in our region, explaining the procedure of transfer. Before working in the MICU team all ICU nurses and intensivists completed a scenario-based training in our skills lab.

Only patients already admitted to the ICU are transferred by MICU. Transfers are performed seven days/week between 8:00 and 24:00. In order to accomplish the transfer, the referring intensivist has to consult the MICU-coordinator, who completes a MICU transport form with patient characteristics and study data. After authorization of the transfer by the MICU-physician and the supervising staff member of the accepting ICU, the MICU sets out to transfer the critically ill patient.

When the MICU-team arrives in the referring ICU, the patient is stabilized and prepared for transfer; if respiratory insufficiency in a non-intubated patient during transfer is to be expected, the patient undergoes intubation. During transport, the MICU-nurse or physician completes forms describing haemodynamic and ventilatory variables.

Although responsible for all performed transfers with MICU in the north-eastern region of the Netherlands, our university-based ICU is not always the ICU of destiny. In this study, we included transports to our ICU and to the ICU of the Scheper Hospital in Emmen.

The following data were collected: blood pressure (systolic, diastolic, mean arterial pressure), heart rate, respiratory rate, body temperature, ICU and hospital mortality; arterial blood gas analysis (saturation, pH, paO_2_, paCO_2_, bicarbonate), lactate, glucose and haemoglobin; mechanical ventilation settings and use of vasoactive or inotropic medication and the presence of a (central) venous or arterial catheter. Data were collected before the moment of departure, on arrival and 24 hours after admission in the referral ICU. Data from blood sampling and other data acquisition on arrival were noted when the patient was settled in the referral ICU-bed.

After arriving at the referral ICU, APACHE II scores (Acute Physiology and Chronic Health Evaluation) were determined for all patients. This score, based on patient scores within the first 24 hours of ICU admission, provides an indication of the severity of illness on which mortality risk can be predicted. Since almost all patients are taken over from other ICUs, in which admission primary APACHE scores are being determined, the listed scores are secondary APACHE II scores.

Since this study concerns an evaluation of a present standard of care, ethical approval and informed consent are not a requirement. The medical ethics committee of our university medical center was informed and approved the design of our study.

### Statistics

We performed a Paired-Sample T-test to evaluate the variables before and after transport. This test is used to determine the equality of means of two related groups. Performing this analysis, each parameter could be tested on significant changes within the period of transportation. Before comparison, 'critical thresholds' were predefined. In order to see whether the distribution of a variable passing a critical threshold differed in time (indicating major deterioration), we performed the McNemar test. This test is used to compare dichotomous variables in a repeated measures situation (where subjects are assessed before and after an intervention). Each variable (whether beyond threshold or not) at departure and arrival could be analyzed this way.

The number of patients in whom a critical threshold was reached during transport was calculated (with normal values on departure but values beyond critical thresholds at arrival indicating a worsening in the patient's condition). To objectify whether there was a difference between departure and arrival concerning total number of variables beyond threshold, we used the Wilcoxon signed ranks test. *P *< 0.05 was considered statistically significant. Data were analyzed using SPSS for Windows 16.0 (SPSS Inc. Chicago, IL, USA).

The critical thresholds are regarded as clinically relevant deteriorations. For instance, the haemoglobin threshold of 4.4 mmol/L (7 g/dl) is being cited in the study by Hébert, in which a restrictive strategy of red cell transfusion within the critical care is recommended [10]. The threshold of the mean arterial pressure (MAP below 60 mmHg) is associated by an increased risk of death in early septic patients [11]. Thresholds concerning body temperature are also being cited in literature [12,13]. Remaining thresholds are based on clinical practice in the Netherlands.

## Results

From March until December 2009, 74 transfers were performed to our university affiliated ICU and the ICU of the Scheper Hospital, Emmen. Characteristics of the transferred patients are summarized in Table 1. All transfers were from 14 regional hospitals in the north-eastern region of the Netherlands. Two ICUs transferred 10 patients or more, four ICUs transferred between five and nine patients and eight ICUs transferred less than five patients. The main indication for transfer was the need for higher intensity of care or advanced therapy; for example, renal replacement therapy. The main diagnosis at transfer was respiratory failure (27%), followed by sepsis (17.6%) and multi-organ failure (10.8%) (Table 2).

**Table 1:** Baseline characteristics of patients

Variables (Percentage)	
No. patients	74
	
Age (years)	
Mean	59.8 ± 15.6
Median	62
	
Sex (female/male)	53/47
	
Mechanically ventilated	84
Oxygen by	
Mask	9
Nasal tube	7
	
Central venous line	76
Intra-arterial catheter	92
Peripheral venous line	87
	
Vasopressor/inotropic drugs	53
	
APACHE II score	20 ± 8.1
	
Duration of transfer (min)	51.3 ± 19.1
	
Reason transfer	
Logistic	20
Advanced therapy	80

APACHE, Acute Physiology and Chronic Health Evaluation.

**Table 2:** Main diagnosis at transfer

Diagnosis (percentage)	
Respiratory problems	27
Sepsis	17.6
Multiple organ failure	10.8
Neurological disorders	10.8
Surgical problems	9.4
Cardiac disorders	8.1
Gastrointestinal bleeding	6.8
Intoxication	2.7
Other*	6.8

*Other diagnoses included pulmonary embolism (*n *= 1), major bleeding after elective conchaesurgery (*n *= 1), Wegener's granulomatosis (*n *= 1), Hemolysis Elevated Liver enzymes Low Platelets (HELLP) syndrome (*n *= 1) and acute renal failure (*n *= 1).

### Incidents

The primary aim of this study was to evaluate the safety of the transportation protocol for critically ill patients. Incidents during transfer are noted in Table 3. In summary, nine incidents were recorded; all of them due to technical problems. As a consequence, two transfers were performed by an ambulance without changing the escorting retrieval team.

**Table 3:** Incidents during MICU transfer

Incident frequency (%)	
Leakage of compressed air	4.1
MICU breakdown, due to	1.4
-dysfunctional loading bridge*	1.4
-dysfunctional exterior lighting*	1.4
-starting problems	1.4
No display respiratory curve	1.4
Failure heater during transfer	1.4
Failure perfusorpump to purge	1.4

**Total**	12.5%

* transfer performed by great volume ambulance.

### Adverse events

In three transfers, a leakage of compressed air was present without indication of shortage of oxygen. In these transfers there was a modest decline in saturation at arrival (92% vs. 96%). During a transfer in which the heater broke down, body temperature of the transferred patient declined from 37.8 to 34.8°C.

Table 4 shows the mean of variables at the moment of departure (last values of the referring hospital), arrival (first values after admission in the referral hospital) and 24 hours after admission. The corresponding T-values are related to values at departure and arrival. It also shows the percentage of patients who had each variable beyond critical threshold with corresponding *P-*values (also between departure and arrival). Recording variables at 24 hours could give an indication of whether a decline during transfer is reversible or if there is a progressive deterioration (or improvement) within the first 24 hours after admission, and, therefore, these are also shown.

**Table 4:** Variables at departure, arrival and 24 hours after arrival in the referral ICU

						Beyond threshold			
								
Variable	Departure	Arrival	24 h after arrival	T*	Threshold	Departure-	Arrival-at	(%) 24 h	P**
Heart rate	98.4 ± 19.2	96.5 ± 19.5	89.4 ± 20.3	0.36	< 50 and >120	0 to 8	0 to 7	0 to 4	1.00
Syst. BP	121.2 ± 19.8	131.1 ± 27.2	121.2 ± 19.4	0.00	< 90 and >180	1 to 1	3 to 7	0 to 0	0.18
Diast. BP	61.6 ± 13.6	64.8 ± 14.1	57.8 ± 12.6	0.07	< 50 and >110	12 to 1	12 to 0	15 to 0	1.00
MAP	80.8 ± 14.0	87.0 ± 16.6	78.9 ± 13.1	0.00	< 60	3	4	4	1.00
Temp (°C)	37.7 ± 1.4	37.6 ± 1.2	37.5 ± 1.1	0.26	< 36 and >39.5	5 to 11	5 to 8	4 to 4	1.00
Resp. Rate	19.3 ± 6.3	20.6 ± 6.6	19.9 ± 6.6	0.12	> 30	4	10	8	0.29
Saturation %	96.0 ± 6.7	95.8 ± 5.7	96.0 ± 6.4	0.76	< 90	5	10	3	0.45
Art. pH	7.38 ± 0.10	7.38 ± 0.11	7.37 ± 0.09	0.90	< 7.30	16	18	15	1.00
PaO_2 _(kPa)	12.2 ± 3.6	14.0 ± 6.7	12.2 ± 3.4	0.76	< 8	4	8	4	0.25
PaCO_2 _(kPa)	5.4 ± 1.3	5.3 ± 1.3	5.6 ± 1.3	0.76	> 6	20	23	28	0.77
HCO_3_^-^	23.6 ± 5.6	23.4 ± 5.5	23.7 ± 5.3	0.99	< 20	18	22	19	0.69
Haemoglobin	6.6 ± 1.3	6.3 ± 1.1	6.1 ± 1.0	0.04	< 4.4	0	1	3	1.00
Lactate	2.6 ± 3.0	1.9 ± 2.8	1.6 ± 1.2	0.15	> 3	11	8	5	0.45
Glucose	7.0 ± 1.5	7.6 ± 2.1	7.4 ± 1.9	0.03	< 4 and >12	1 to 0	1 to 4	1 to 1	0.38

* T-values were calculated using Paired Samples T-test (between departure and arrival).

** *P-*values were calculated using the McNemar test (between departure and arrival).

Heart rate and respiratory rate is beats per minute respectively breaths per minute. Systolic, diastolic blood pressure (BP) and MAP (mean arterial pressure) are expressed as mmHg. Laboratory values (bicarbonate, haemoglobin, lactate and glucose) are expressed as mmol/L.

PaO_2, _partial arterial oxygen tension; PaCO_2, _Partial arterial carbon dioxide tension.

The median number of passed critical thresholds was one at departure, as well as at the moment of arrival. Maximum number of variables beyond a critical threshold was seven after transfer versus five before transfer. The total number of variables beyond threshold before transfer was compared to the number of variables beyond threshold at arrival, a greater number of variables at arrival indicating deterioration of the transferred patient.

**Figure 1:**
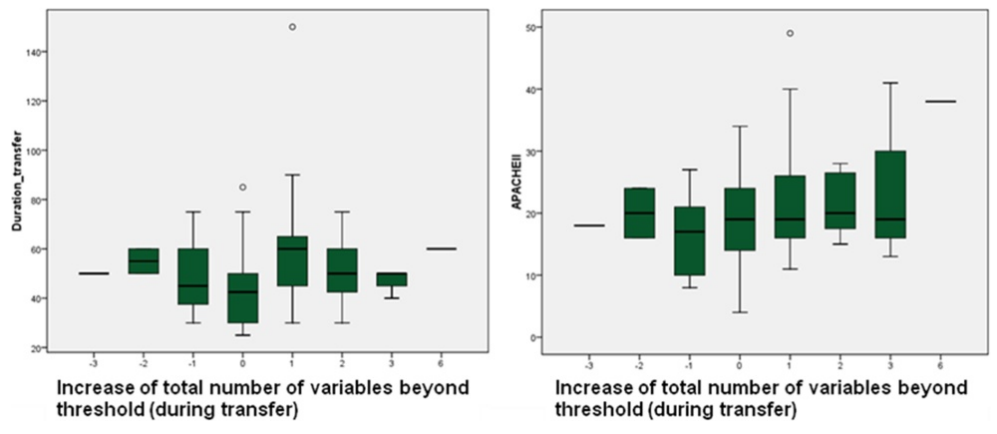
**Distribution of duration transfer (minutes) and APACHE II score**.

Table 5 shows the change in total number of variables beyond critical threshold during transfer. Analyzing patient groups based on a decrease or increase of deteriorated variables did not reach statistical significance (*P *= 0.11 by Wilcoxon Signed Ranks test). Analyzing transportation time of patients who showed a decrease or increase of the total number of variables beyond threshold did not show significant variance (*P *= 0.20 by One-Way ANOVA), nor in severity of disease (APACHE II; *P = *0.11 by One-Way ANOVA). Figure 1 shows the distribution of these two dependent variables.

**Table 5:** Course of total number variables beyond threshold during transfer

Total variables beyond threshold (after transfer)	Percentage of patients	Number of incidents
Decrease	30*	2
Equal	32*	5
Increase of	38*	2
*1 variable*	*23*	*2*
*2 variables*	*10*	-
*3 variables*	*4*	-
*4 variables*	*1*	-

* P = 0.11 by Wilcoxon Signed Ranks-test.

### Arrival in the referral ICU

Within 24 hours of admission four patients died (5%) and three patients (4%) were already transported to a normal care unit. Mean number of passed critical thresholds of the deceased patients before transfer was 2.3, at arrival this was increased to 3. One patient had one less variable beyond threshold after transfer, three patients showed an increase of respectively one and two variables beyond threshold. Mean APACHE II score was higher in the group of deceased patients compared to the remaining patients, although the difference did not achieve significance (34.7 vs. 19.8, *P = *0.13 by Independent-Sample T-test; one APACHE-score missing). One patient died because of a newly diagnosed ruptured thoracic-abdominal aortic aneurysm, one patient died during asystole in which resuscitation was not successful, the two other patients were abstained because of no therapeutic options in severely diffuse brain ischemia and severe metabolic acidosis refractory to therapy. ICU mortality of the study population was 28%.

### MICU vs. standard ambulance transport

Data from the study on interhospital transfer by standard ambulance in 2005 enables comparison of transfer and impact on patient stability during transfer. In the transfers by MICU, as for patients transferred by ambulance, the main diagnosis at the moment of transfer concerned respiratory problems (32% by ambulance vs. 27% by MICU), sepsis (10% by ambulance vs. 17.6% by MICU) and multi-organ failure (25% by ambulance vs. 10.8% by MICU). Due to the observational study design, it is not possible to compare patient groups directly, but to evaluate the effects of this new transportation mode we analyzed incidents and patient stability in the following way:

In 2005, incidents were recorded in 34 transfers (34%), of which 30% was related to technical failure and 70% was due to transfer organization or staff management. It was estimated that in this latter group, up to 50% could have been prevented by better preparation before transfer. In transfers by MICU, the incidents related to technical failure are comparable to technical failure by ambulance (12.5%), but in the present situation we did not find incidents related to staff management or inadequate preparation.

**Figure 2:**
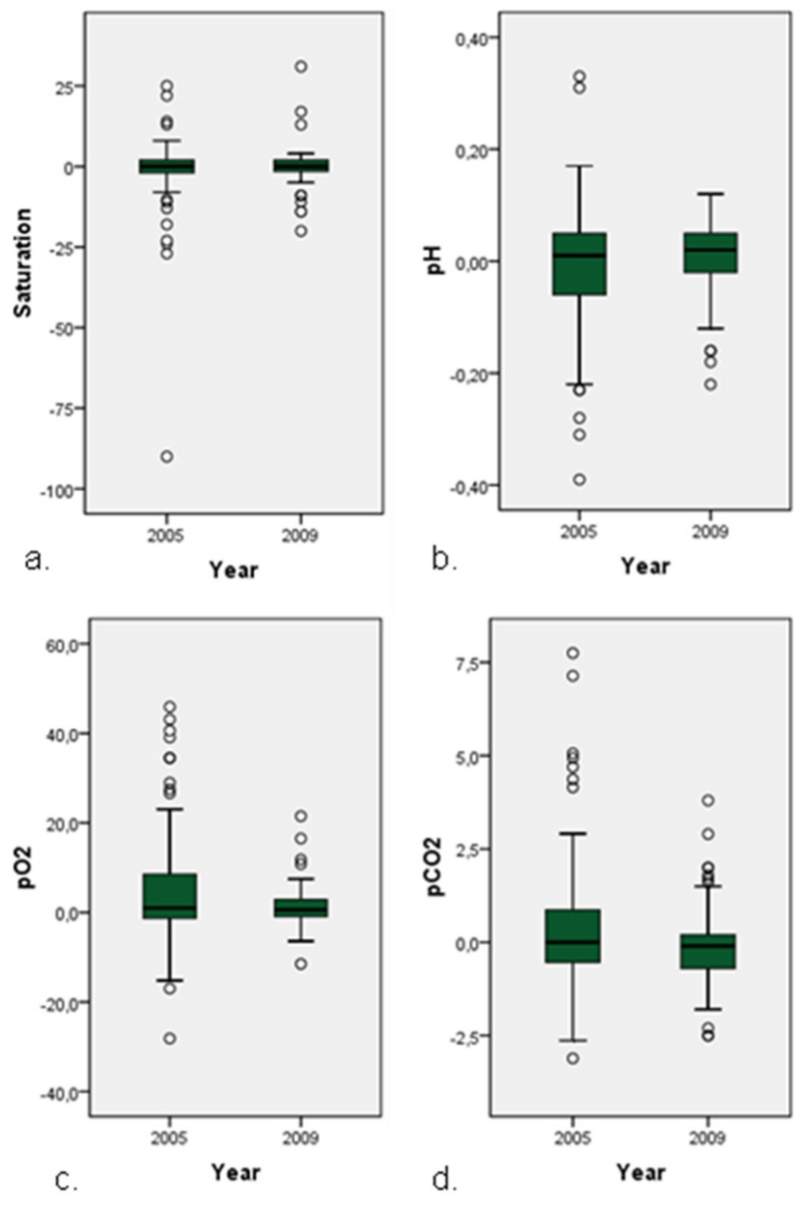
**Individual course of arterial blood gas analysis during transfer by ambulance (2005) and MICU (2009)**. **a**. Differences in saturation (expressed as percent), *P = *0.16*. **b**. Differences in pH (expressed as mmol/L), *P = *0.02*. **c**. Differences in pO_2 _(expressed as kPa), *P = *0.00*. **d**. Differences in pCO_2 _(expressed as kPa), *P = *0.02*. * by independent-samples.

Analyzing patient stability during transfer by ambulance, statistical significance in vital variables was not present. Deterioration of pulmonary status, however, was prominent: with standard ambulance transfer, at arrival in the referral hospital five patients required imminent mechanical ventilation. With MICU-transports, this did not occur. To visualize respiratory status during both ways of transfer, arterial blood gases were analyzed. The course of these variables from both years is displayed in Figure 2. Distribution of differences in arterial blood gases during transfer in 2009 versus 2005 showed significant better values for the variables pH, paO_2 _and paCO_2 _in the patient group transferred by MICU, using the Independent-Samples T-test (α <0.05).

## Discussion

Assessing safety of transport of critically ill patients, primary endpoints in this study were patient status (expressed in 14 vital variables), incidents, adverse events and interventions during or shortly after interhospital transfer by MICU. Data from interhospital transfer by standard ambulance transport, as performed in 2005, were compared to data of the current study.

In 12.5% of all transfers performed by MICU, there was a technical failure which showed little impact on patient status. Incidents due to staff (mis)management were not present and unlike 2005, no interventions have been necessary during or shortly after transfer by MICU in order to stabilize the transferred patient. In summary, the number of incidents have been reduced to a minimum compared to 2005 and might even improve after gaining experience with the MICU device.

Evaluating the course in patient status during transfer, systolic blood pressure (and mean arterial pressure), haemoglobin and glucose were significantly different at the moment of arrival. The increase in systolic blood pressure (121.2 to 131.1 mmHg, *P = *0.00), however, may indicate an altered hemodynamic circulation rather than a major deterioration. Blood glucose level increased from 7.0 to 7.6 mmol/L (*P = *0.03) as the haemoglobin concentration declined from 6.6 to 6.3 mmol/L (*P = *0.04). These variables are significant changes, but the clinical relevance seems dubious. We therefore predefined critical thresholds in each vital variable to objectify major deterioration. Analyzing variables passing critical thresholds during transfer by the McNemar test, we did not obtain significant major deterioration in the MICU-patients.

Another way of indicating deterioration is to evaluate individual data on total number of variables beyond a critical threshold at the moment of departure and arrival. Patient groups, having a decrease or increase in total number of passed critical thresholds, were not significantly different from each other. Furthermore, the group of patients having an increased number of variables beyond threshold at arrival did not have significant longer transportation time, nor did they suffer more incidents during transfer.

Risk of mortality is predicted on the severity of disease, expressed as an APACHE II score which is calculated within the first 24 hours after admission. As most patients are transferred from other ICUs, our APACHE II scores are secondary scores. Prior stabilization in referring ICUs may, therefore, underestimate Standardized Mortality Ratio (SMR) predicted by our secondary APACHE scores. Mean APACHE II score of our study population was significantly higher than the APACHE II score of our total ICU population: 20.0 vs. 14.5 (*P *< 0.001 by One-Sample T-Test). ICU mortality was 28% in our patient population, which is also higher compared to the mortality (8.8%) of our total ICU population.

Like the patients transferred by ambulance, data on transferred patients by MICU show a stabile course in patient status. However, despite the absence of significant major deterioration in patient status in 2005, some patients were respiratory insufficient and needed imminent intubation on arrival at the referral centre. Displaying the course of pulmonary parameters during both ways of transfer, patients transferred by MICU show less deterioration compared to the transferred patients in 2005.

The safety of a specialist retrieval team with or without a Mobile Intensive Care Unit is also found in the article of Bellingan *et al. *[8] and there are more studies that emphasize the importance of a well-established transfer protocol [9,14]. Similar to these studies, we do not have any data of patients who were not transported. It is, therefore, not possible to state that transfer is beneficial to patient survival. However, the transferred patients had a higher APACHE II score than our general ICU population, which gives the impression that the way of selecting patients for referral is adequate. When looking at our data, transfer by MICU appears to be safe despite the high degree of severity of disease. We, therefore, conclude that the safety of the current way of transporting the critically ill is warranted and that the MICU sets a major improvement in quality of care for the critically ill.

## Conclusions

Initiating interhospital transport involves deliberation of various determinants such as patient status, transfer indication, escort and transport facilities. The MICU has gained a role in the national guideline concerning interhospital transfer of critically ill patients. This observational study of MICU transfer shows that transfer by MICU is not associated with major deterioration in patient status and that the implementation of a transport protocol with a mobile Intensive Care Unit has led to an improvement in quality of care on the road, compared to the former way of transfer.

## Key messages

• From 2009 on, interhospital transfer has been performed by a Mobile Intensive Care Unit with a specialized retrieval team according to national ICU guideline and law.

• Patient status during MICU-transfers showed no major deterioration in any of the vital variables, despite a high severity of disease (expressed as the APACHE II score).

• All incidents occurring during MICU-transfers were related to technical failure and were shown to have little influence on patient status.

• Excessive deterioration in pulmonary status is not present in the MICU-transfer and has, therefore, shown improvement in the support of respiratory status before and during transfer compared to transfer by standard ambulance.

## Abbreviations

Adverse event, unintended injury related to medical management (or transfer); APACHE II: Acute Physiology And Chronic Health Evaluation; DBP: diastolic blood pressure; FiO_2_: fraction of inspired oxygen; HELLP: Hemolysis, Elevated Liverenzymes and Low Platelets-syndrome; ICU: intensive care unit; Incident, unintended event which may have or did reduce the safety margin for the patient; MAP: mean arterial pressure; MICU: mobile intensive care unit; PaCO_2_: partial arterial carbon dioxide tension; PaO_2_: partial arterial oxygen tension; SBP: systolic blood pressure; SMR: standardized Mortality Ratio.

## Competing interests

The authors declare that they have no competing interests.

## Authors' contributions

JSW set up the design of the study, performed data acquisition, carried out data analysis and drafted the manuscript. JMD participated in the design of the study and provided information about transportation protocols and drafted this part of the manuscript. JGZ set up the study design and helped format the statistical analysis. JF helped with the acquisition and interpretation of data. JJML set up the study design and revised the manuscript and has given final approval of the version to be published.
